# Ethnic leadership and the politics of legitimacy: electoral practices in the Chinese diaspora in Thailand

**DOI:** 10.3389/fsoc.2026.1774402

**Published:** 2026-03-27

**Authors:** Zheng Gao, Pharatt Run, Yaoping Liu

**Affiliations:** Department of Management Science, Rajamangala University of Technology Krungthep, Bangkok, Thailand

**Keywords:** Chinese diaspora, community elections, diaspora politics, ethnic leadership legitimacy, governance, race and ethnicity, social integration, Thailand

## Abstract

**Introduction:**

The legitimacy and election processes of community leaders differ across nations due to cultural and ethnic influences. Despite their integration, Chinese community leaders in Thailand face challenges in gaining legitimacy. The study emphasizes how ethnic and culture impact leadership selection and acceptance, reflecting broader societal values and structures.

**Methods:**

This study employs qualitative methods to understanding of Chinese diaspora perception and experience on electoral process. Semi-structured interviews were conducted with five Chinese diaspora and two Thai policy makers. This study used phenomenological techniques for its data analysis. Understanding these variations provides insight into the complexities of leadership legitimacy in multicultural societies.

**Results:**

This study found that Chinese diaspora are aware of the government and organization rules and issues surrounding electoral process and have provided comprehensive insight on the solution to these issues.

**Discussion:**

This research contributes to discussions on leadership, cultural identity, and social integration, offering a comparative perspective on how community leadership functions across diverse cultural landscapes.

## Introduction

The concept of leadership legitimacy is crucial for comprehending the process of leader election and recognition within communities (Junaidi, 2025; Mexhuani and Mexhuani, 2023; Wuyts et al., 2026). The elections have become a primary process for acquiring legitimacy for leadership. The election process enables community members to manifest their enthusiasm for future leaders, thus providing elected leaders with a robust foundation of legitimacy based on the majority’s support. The legitimacy and election processes of the Chinese diaspora in Thailand also become a complex issue because they are influenced by the long establishment of historical, economic, and political ties between the two countries (Camba, 2023; Murphy, 2017; Thunø and Wang, 2025). The legitimacy of Chinese leadership in this context often hinges on how these initiatives are perceived by the Thai population and political elites based on the cultural contexts. Ethnic diversity influences legitimacy in Malaysia and Indonesia, where the Muslim population is dominant. Malaysian leaders gain more legitimacy if they are Malay, while Indonesia’s ethnic diversity creates more complex leadership dynamics (Latif, 2024; Sealy et al., 2022). In Thailand, where Buddhism is prevalent, legitimacy follows a different pattern. The ethnic Chinese community, though integrated into Thai society, faces challenges in gaining leadership legitimacy toward balancing their cultural heritage with Thai national identity (Chachavalpongpun, 2024; Raymond, 2020).

The elections in the context of Chinese community leadership in Thailand serve as a means to validate and establish authority both from the higher levels of leadership (top-down) and from the grassroots (bottom-up) level (Huque and Jongruck, 2020; Wongpreedee and Sudhipongpracha, 2024). The top-down method means that state authorities in the community recognize and support the idea of authentic leadership. These authorities often set standards and requirements for what real leadership looks like. In contrast, bottom-up procedures involve the active involvement and agreement of community members themselves, who desire leaders capable of effectively representing their interests and goals. These two methods contribute to the establishment of enduring legitimacy for reciprocal relationships (Bernhard et al., 2020; Martin et al., 2021). Transparent and participatory elections enhance the confidence of community members, while endorsement from higher authorities guarantees that leadership is formally acknowledged and can operate efficiently within established legal and social structures (Al Meslamani, 2024; Siddiq and Ahmed, 2026). However, the legitimacy of these structures can be weak and prone to internal conflict if community members do not actively participate and agree with them, leading to potential challenges in governance and a lack of trust in the decision-making processes, which may result in decreased community engagement and increased dissent against leadership.

While prior research has explored various dimensions of the leadership process and attributes within the Chinese diaspora, the concept of legitimacy remains an insufficiently examined personal process among leaders. Furthermore, a comprehensive review is necessary to assess the complexity of the legitimacy and election procedures for Chinese community leaders, particularly in Thailand, given its multicultural environment (Anthony, 2023; Khuenkhaew et al., 2025). Revisiting the legitimacy and election process of leaders has become a central area of study in numerous nations. It has been acknowledged that a transparent and participatory election process plays a crucial role in enhancing community trust in local leaders (Haryanto et al., 2019; Schugurensky and Mook, 2024). Conversely, research conducted in India on village head elections in rural areas demonstrates that the legitimacy of leadership can be enhanced by implementing an election process that is inclusive and fair (Kumar, 2021; Menon and Rassendren, 2022). The Thai government has an intricate electoral procedure, frequently incorporating a blend of selection and promotion (Fossati, 2025; Sawasdee, 2023). This demonstrates the intricate political and social dynamics present in Thailand, which in turn leads to a growing complexity in the selection process of Chinese community leaders in the country.

The concept of legitimacy and the electoral procedure can enhance leadership positions and guarantee that individuals elected have strong and legitimate support from their communities (Drew and Kim, 2025; Sheng et al., 2025). Furthermore, some evidence suggests that the electoral process effectively legitimizes leadership. A lack of study investigates the key success of leadership transformation among community members, specifically in the developing countries. How does the process for electing and determining the legitimacy of Chinese community leaders in Thailand work? How does the legitimacy and election process for Chinese community leadership differ between Thailand and other overseas countries? This study integrates preexisting methodologies, namely reviews with conceptual, regulatory, survey, or interview methodologies, and incorporates an analysis of implementation techniques.

Examining the impact of societal, political, and technological factors on the legitimacy of Chinese community leadership in Thailand offers an explanation of how external factors influence community dynamics. This aspect is essential for comprehending the adaptability and resilience of the Chinese diaspora in the evolving contexts of host nations. The findings of this study can have practical implications for policymakers, community leaders, and members of the Chinese community in Thailand and other Southeast Asian countries. A deeper understanding of the foundations of leadership legitimacy and the factors affecting it can guide improvements in community governance and conflict resolution, particularly by addressing the unique challenges faced by the Chinese diaspora in Southeast Asia.

## Literature review

### Legitimacy theory

Legitimacy theory in the election process is a crucial concept for explaining the basic principles under the umbrella of democracy (Camba, 2023; Schmidt and Wood, 2019). This theory postulates that elections provide a systematic mechanism through political power and law (Thurston, 1975). The legitimacy of election processes relies on some elements, including fairness, inclusivity, and transparency. When these key elements are upheld, the election process and outcome will contribute to legitimation and representation of the community and society (Fossati, 2025; Weber, 1968). For instance, in the establishment of democracies like the Asian, United States, and European countries, adherence to the legitimacy of electoral laws, commissions, and robust mechanisms for voter participation contributes to the credibility and legitimacy of election processes and outcomes (Weber, 1968). The significance of legitimacy lies in its ability to establish the confidence and credibility of an institution on a global scale (Smith, 2021).

Legitimacy in the context of the Chinese diaspora refers to the perceived rightful authority and acceptance of governing institutions (Dodworth and Hönke, 2026). This process is an effective governance to underpin diaspora members to recognize and comply with the decisions and policies, whether these organizations are linked to the Chinese state or local authorities (Chen, 2021). However, effective legitimacy depends on transparency, accountability, and responsiveness to the diaspora’s social, economic, and political needs (Kumar, 2021; Smith, 2021), which means that without these elements, the governing institutions may struggle to gain the trust and support of the diaspora community. Moreover, legitimacy can be influenced by the relationship between the Chinese state and its diaspora, which includes the state’s efforts to engage with and support overseas Chinese populations (Han and Khemanitthathai, 2022; Thunø and Wang, 2025), as well as the diaspora’s perception of these efforts and their effectiveness in addressing their needs and concerns. It demonstrates that legitimacy in Chinese diaspora governance is the foundational element that enables governing entities to maintain authority, foster community cohesion, and facilitate cooperation between diaspora members and the local people.

### Chinese community and leadership in Thailand

Chinese overseas communities in Thailand have significantly influenced the country’s socio-economic and cultural values. Their presence over several centuries has become a cornerstone in the development and diversification of Thailand (Da Wan et al., 2020; Song and Wang, 2024). The leaders are in charge of some important areas, such as business, politics, and preserving culture. They also stress how important the community is to Thai society, as it fosters social cohesion, supports local economies, and preserves cultural heritage. Chinese immigrants in Thailand have a long-standing reputation for entrepreneurship. This spirit has been a driving force behind their successful integration into Thai society with regard to establishing a diverse array of businesses, from small family-owned shops to large-scale corporations (Caballero-Anthony, 2022; Somwethee et al., 2023). The economic contribution of these businesses is profound, spanning various sectors and creating a ripple effect that boosts the Thai economy. Their success stories serve as an inspiration and a bridge between cultures, fostering a deeper understanding and appreciation of Chinese heritage within Thailand (Zheng and Liu, 2024; Cormoș, 2022).

## Methods

### Research design

This study used a basic qualitative approach to collect and analyze data to answer the research questions, which were meant to examine how Chinese diasporas develop careers, as in Thailand. A basic qualitative approach is interested in (1) how the Chinese diaspora explains their experiences and (2) how participants develop their organizations (Fan et al., 2025). In qualitative research, the researcher is the main instrument for collecting and analyzing data. An interview guide was created for the purpose of conducting in-depth interviews, which were an opportunity to explore the challenges facing the Chinese diaspora. The interviews were recorded using a voice recorder to ensure that rich data were obtained. The data were then transcribed verbatim and analyzed into groups so that themes could be identified (Creswell and Creswell, 2022). A qualitative approach was selected for this study because it is beneficial for studies seeking to reflect leadership legitimacy and electoral processes among the Chinese diaspora in Thailand. It enables a researcher to see how people interpret their experiences, construct the world, and associate meanings with it (Lim, 2024). A qualitative approach also facilitates the generation of comprehensive insights regarding leadership legitimacy as perceived by the Chinese diaspora in Thailand, along with their experiences.

### Research setting and participant recruitment

Information about the participants was obtained through organizational member data provided directly by the organization. The informants were selected from different organizations. A purposive sampling approach was used to focus on the specific population group relevant to the research topic. The sample consists of well-educated and articulate individuals who can understand and respond to detailed questions about specific issues related to the Chinese diaspora in Thailand. Creswell and Creswell (2022) recommend that seven interviewees for in-depth exploration in phenomenological studies are sufficient to identify key themes. Participants had to meet the following eligibility criteria: Participants had to (1) have run an organization for a minimum of two years; (2) be between 30 and 50 years old; and (3) be capable and willing to participate in this study.

### Data collection

After selecting and contacting participants, and prior to the actual interview session, we met with the informants to introduce ourselves and to build a relationship so that we could get some information about the Chinese diaspora activities in Thailand conducted by the informants. This procedure was done to build a sense of familiarity or rapport with the informants so that they would feel more comfortable sharing information in the actual interview sessions. The interview began with a question asking the informant about their background as Chinese diaspora and Thai policymaker. At the interviews themselves, the goals of the study and how the interviews would be conducted were explained to the informants. Face-to-face interviews were conducted by two team members; one facilitated the interviews, and another one acted as a note taker, documenting observations and reflections. Data collection and transcription were conducted simultaneously to create more opportunities for revising or prompting new questions. After seven interviews were conducted, no new information was presented, and data saturation was reached. Each participant was interviewed once, as we found that sufficient data had been gathered to support the key themes and topics in detail. All interviews were transcribed verbatim.

### Data analysis

Firstly, the researchers familiarized themselves with the original database (Horton et al., 2004). Then, line by line, initial comments and codes were made to form the initial codes by an experienced researcher. Then the initial codes were identified and grouped initial emerging themes. At this stage, two researchers checked the reliability by going back to the original data and matching the initial codes with each coded transcription. After all the transcriptions were analyzed, all the coded data were grouped to identify the patterns and connections by using a thematic map. Finally, a table was developed to present and compare the emerging themes across all the transcripts, identifying and consolidating the final themes. To ensure rigor, the findings were sent back to participants for member checking, allowing them to confirm that the themes accurately represented their experiences. The research team also frequently met and discussed any issues raised during the analysis process, ensuring a consistent outcome.

Data saturation was obtained when constant techniques were used. The constant comparative method involves continuously adding new cases to datasets up to the saturation point (Lim, 2024). It consists of four steps: (1) an interview session, (2) transcription of the interview, (3) open coding, and (4) obtaining confirmation from the informant. In the third step, the open coding of the transcript was compared with that of the previous informant. This method allowed for the determination of data saturation by comparing the open coding in this manner. When there was repetition and no new information emerged, the data saturation point had been reached. In this study, data saturation was reached after the researcher compared the open coding and grouped the data into categories and themes based on the time and other resources available for the research. Seven interviewees from three different backgrounds with different exposure, insight, and experience were chosen for this study, as shown in Table 1.

**Table 1:** Interviewee profile.

No	Initial name	Education	Position
1	Mr. LK	Master	Chinese community leader
2	Mr. CL	Master	Chinese community leader
3	Ms. LD	Master	Chinese community
4	Mr. PT	Ph.D	Chinese community
5	Ms. WD	Ph.D	Chinese community
6	Ms. LH	Ph.D	Thai policy maker
7	Mr. YP	Ph.D	Thai policy maker

## Results

Theme 1: Organization commitment to law and society

This theme reflected how the organization’s arrangement and its members respect government rules. The theme also reflected the participants’ experiences and expectations following their stay in the care home. This theme emerged from interview responses and document analysis highlighting the Chinese community in Thailand’s active participation in understanding, complying with, and promoting awareness of local policies. The theme was identified through repeated references to seminars, conversations, and government engagement aimed at educating community members about Thai regulations. For example, participant 1 emphasized a collective commitment to policy adherence as essential for maintaining positive relationships with local authorities and communities.

In general, our community follows the policies in place. We always aim to comply with local rules and regulations, and we offer seminars and conversations to ensure that community members understand the relevant policies (Mr. LK).

In the same context, interviewee Ms. LD emphasized the importance of community and government role functions because the existence of excessive policies can be considered the main rules. The rule is also consistent with the objectives of the community and government, interviewee Ms. LD said:

I constantly attempt to be the connection between the community and the government. We hold regular conversations and encourage government officials to communicate directly with our communities, and we follow existing policies in Thailand. I also believe the level of compliance is fairly high. Most of us understand that following policy is for the common benefit and preserving good ties with the government.

However, some participants noted challenges, especially with newly enacted policies. Ms. WD highlighted this gap:

I believe that most individuals of our community strive to follow applicable policies. We understand that this compliance is critical to maintaining positive relationships with local communities and governments. However, some of us may not fully comprehend all of these policies, particularly those that have just been enacted. We hope for further outreach and support by the government to help us understand and comply with the regulation better.

The Chinese diaspora organization enlightens its members about new Thai regulations through workshops and focus group discussions to mitigate miscommunication between community members and these regulations.

Our organization organizes regular workshops and training for all members, focusing on Thai legal requirements and ethical standards. We also conduct internal audits to detect and correct any potential issues early. Demonstrating a strong commitment to legal compliance reinforces our legitimacy and helps us integrate into Thai society (Mr. CL)."

Similarly, participant 6, as a Thai policymaker, expressed that the Chinese diaspora organization and members care about Thai regulations. Therefore, she has no concerns about the organization community, which reflects her satisfaction and happiness because the community has a pivotal effect on Thai people.

The organization and members respect regulations and maintain transparent documentation and reporting systems that are open for inspection by relevant Thai authorities, which strengthens the organization's reputation and legitimacy (Ms. LH).

To mitigate the miscommunication between community members and new Thai regulations, the Chinese diaspora organization enlightens the members about new regulations with regard to workshops and focus group discussions.

Our organization organizes regular workshops and training for all members, focusing on Thai legal requirements and ethical standards. We also conduct internal audits to detect and correct any potential issues early. Demonstrating a strong commitment to legal compliance reinforces our legitimacy and helps us integrate into Thai society (Mr. CL).

Similarly, participant 6, as a Thai policymaker, expressed that the Chinese diaspora organization and members care about Thai regulations. Therefore, she has no concerns about the community organization, which reflects her satisfaction and happiness, as it plays a crucial role in fostering positive relationships and collaboration with local authorities for Thai people.

The organization and members respect regulations and maintain transparent documentation and reporting systems that are open for inspection by relevant Thai authorities, which strengthens the organization's reputation and legitimacy (Ms. LH).

Theme 2: Transparency in the selection process

This theme originated from detailed descriptions of the election procedures for Chinese community leaders in Thailand, emphasizing transparency, inclusivity, and government oversight. Participants consistently depicted elected officials as trustworthy representatives who conform to cultural norms and community standards. In Thailand, the process of electing a Chinese community leader entails several processes, such as forming the community, ensuring transparency, and involving the government. Transparency is attained by implementing several measures, such as verification procedures, impartial supervision by local governmental entities, and public validation. The process entails the active involvement of all communities, who contribute by disseminating information about the candidates and their respective visions prior to the election. Subsequently, this data is utilized to make well-informed judgments. The government plays a facilitating role in the process, as it establishes an independent committee to supervise the election process. In this regard, interviewee Mr. PT, a Chinese community leader, stated that:

Community members vote to determine the election results. Any eligible member may vote or run for leadership. I believe the process is pretty transparent. All election stages are publicly disclosed, and an independent election body oversees the process. Furthermore, we consistently have clear information on the candidates and the selection process.

In the same context, interviewee Ms. LH emphasized the importance of the selection leader process because the existence of the community depends on the transparency of the leaders’ selection process.

From our perspective, the process of selecting leaders for the Chinese community in Thailand is well-structured and open. The Chinese community has a defined electoral procedure, which includes oversight from an impartial body to assure fairness. This supports Mr. YP's assertion that general elections among community members often select Chinese community leaders.

Chinese community leaders are often selected through general elections among community members. The government also supervises the election process to guarantee that it is fair and transparent. Transparency is maintained in multiple ways. First, all candidates and the election committee must adhere to the election regulations. Second, the government monitors to verify that there is no fraud or violation. Election results are also made public and available for verification.

Additionally, interviewee Ms. WD evidences that the practice of Chinese community management in Thailand is valuable to build reciprocity relationships:

The election is conducted via direct votes from community members. Every member of the community is welcome to suggest themselves or candidates they believe are capable. This method is completely transparent, and every vote is counted honestly.

The process of selecting Chinese community leaders is conducted in accordance with the regulations established for the Chinese community and adheres to the current regulations in Thailand. This determination is based on document analysis and interviews. The selection of Chinese community leaders follows the constitutional framework of Thailand; Chinese community leaders do not undergo the same general election process as government officials or members of parliament. The findings of this document analysis are corroborated by the outcomes of interviews conducted with Chinese community leaders, Chinese community members, and Thai policymakers. Mr. LK and Ms. WD stated:

In general, our community follows the policies in place. We always aim to comply with local rules and regulations, and we offer seminars and conversations to ensure that community members understand the relevant policies (Chinese community leader).

I believe that most individuals of our community strive to follow applicable policies. We understand that this compliance is critical to maintaining positive relationships with local communities and governments. However, some of us may not fully comprehend all of these policies, particularly those that have just been enacted. We hope the government will provide further outreach and support to help the Chinese community better understand and comply with the regulations.

Theme 3: Legitimacy and community trust.

This theme emerging from combined insights in interviews and document analysis reflects the legitimacy of Chinese community leaders, grounded in transparent election processes, adherence to regulations, and community trust. Participants consistently portrayed elected leaders as credible representatives who align with cultural values and community expectations.

Elected leaders' legitimacy is often high since the election process is transparent and participatory. Most community members believe elected officials actually serve their interests. In general, the community responded positively to the election results. There is mutual trust and respect for the democratic process that has been implemented. If there is dissatisfaction, it is normally addressed through discussion and mediation, which helps to maintain the positive atmosphere and reinforces the community's commitment to constructive dialogue and collaboration (Mr. PT).

We have a lot of respect for those who are elected because the election process is transparent and inclusive. The community generally welcomes the election results and is eager to collaborate with the new leader (Ms. LD).

I believe the elected leaders have strong legitimacy because they were selected in a fair and transparent manner. As community members, we hold the belief that our voices receive respect and consideration. The community's reaction to the election results was mostly positive. We feel that elected officials are capable of representing our interests and carrying out their responsibilities effectively. Usually, we limit and resolve internal dissatisfaction (Mr. YP).

Evidence suggests that this selecting process downplayed the role of family. Every candidate is required to fulfill stringent criteria and undergo an impartial verification procedure to guarantee their selection is based only on their competence and integrity, rather than any familial or personal connections. A separate selection committee is responsible for supervising the entire procedure to guarantee the absence of favoritism or nepotism. The findings of this document analysis align with the results of interviews, indicating that Chinese community leaders in Thailand possess significant legitimacy.

## Discussion

These findings highlight three interconnected themes: organizational commitment to law and society, transparency in leadership selection, and legitimacy and community trust that collectively underpin the governance of Chinese diaspora organizations in Thailand. Firstly, organizational commitment to law and society emerges as a cornerstone of community integration and harmonious relations with the host country. The repeated emphasis on compliance with Thai regulations and proactive engagement with government authorities reflects a shared understanding among community leaders and members that adherence to local laws is essential for social acceptance and organizational legitimacy. As participants noted, regular seminars and workshops are organized to educate members about new policies, demonstrating a proactive approach to mitigating misunderstandings and fostering a culture of legal compliance. This aligns with Al Meslamani (2024), who observed that policy adherence enhances public management and reduces risks, while Drew and Kim (2025) highlight the role of social participation in shaping effective government policy. However, some participants pointed out the challenges of keeping up with newly enacted policies, indicating an ongoing need for government outreach and tailored support.

Secondly, transparency in the selection process for community leaders stands out as vital for sustaining trust and credibility within the community. The election of leaders through open, participatory processes characterized by independent oversight, public validation, and equal opportunity for all members reinforces the legitimacy of those in positions of authority. This is in line with earlier research that showed that the process of changing leaders and holding elections should be well-organized and overseen by the government in order to be seen as legitimate (Gierlich-Joas et al., 2020; Yue et al., 2019). The community’s commitment to fairness and openness not only fosters trust among members but also assures Thai authorities of the organization’s integrity. It sets a benchmark for other migrant and community members seeking to strengthen their internal governance structures and public legitimacy. Transparent and participatory leadership selection processes can serve as a model for building trust, reducing internal conflict, and promoting effective representation. These organizations help bridge cultural and regulatory gaps, facilitating smoother integration and collaboration with local authorities with regard to aligning community practices with host-country legal and ethical standards.

Finally, the findings regarding transparency in leadership selection and the resulting legitimacy and trust have important implications for both the Chinese diaspora in Thailand and broader contexts of multicultural community governance. When leadership is chosen through open, fair, and inclusive procedures with independent oversight and public validation, community members are more likely to perceive the process as legitimate, thus fostering unity and active engagement (Han and Khemanitthathai, 2022; Moreno, 2024). It enhances the credibility and stability of the organization and also promotes a shared sense of purpose. Secondly, the integration of transparent governance practices bridges the diaspora and local people. This alignment reassures Thai authorities of the community’s integrity, potentially leading to stronger institutional support, enhanced cooperation, and improved social acceptance (Chachavalpongpun, 2024; Dodworth and Hönke, 2026). The study suggests that active oversight and support from government bodies are crucial in maintaining procedural fairness and organizational legitimacy. It demonstrates the value of competence, integrity, and inclusivity in leadership, transcending personal or familial ties. It also ensures that leaders are genuinely representative and accountable to their members, which fosters a sense of belonging and encourages active participation in decision-making processes. Internal mechanisms for addressing dissatisfaction such as mediation and open dialogue further consolidate trust, enabling the community to resolve conflicts constructively and maintain cohesion.

## Conclusion

Although some community leaders were elected through familial relations, the process was based on adherence to regulations and fulfillment of requirements, which ensures that leadership is not solely determined by family ties but also by merit and community standards. The situation demonstrates a parallel trend observed in the case of Chinese communities in Thailand, where they have managed to preserve their cultural identity. This underscores how the existing social context and dynamics can alter legitimacy within social and organizational environments. Within the Chinese diaspora, identity politics play a crucial role in electoral participation, as community leaders navigate dual influences from host and home countries, which can affect their strategies for mobilizing voters and addressing community needs, particularly in balancing cultural preservation with the demands of integration into Thai society. Organizational commitment to law and society is a key part of bringing people together and getting along with the host country. The strong emphasis on compliance with Thai regulations and proactive engagement with government authorities demonstrates a shared understanding among community leaders and members that adherence to local laws is vital for social acceptance and organizational legitimacy. For the community to keep its trust and credibility, the process for choosing community leaders must be open and clear. Open, participatory election processes characterized by independent oversight, public validation, and equal opportunity reinforce the legitimacy of community leaders. Furthermore, it serves as a model for other foreigner communities, promoting effective representation, reducing internal conflict, and facilitating integration with local authorities through alignment with host-country legal and ethical standards. Promoting transparency and procedural fairness enhances the critical importance of legitimacy and community trust. This legitimacy enhances organizational stability and credibility, while transparent governance bridges cultural divides and reassures host authorities, potentially resulting in stronger institutional support and greater social acceptance.

### Theoretical contributions

Leadership legitimacy is a foundational concept in political science and organizational studies, shaping governance, authority, and electoral dynamics. Since 2020, scholars have expanded theoretical frameworks to better understand the intersection between leadership legitimacy and electoral processes, particularly within diaspora communities. Legitimacy theory provides a foundational lens for understanding how leaders derive authority and maintain political support. Identity politics play a crucial role in electoral participation within the Chinese diaspora. This theory underscores how leaders gain legitimacy by aligning with cultural values and leveraging nationalistic rhetoric to mobilize voters across transnational networks. Transnational political actors, such as expatriate politicians or foreign-funded community organizations, influence electoral processes through promoting policies that resonate local people and diaspora communities.

### Practical implications

Understanding leadership legitimacy and electoral processes has several practical implications for governance, policy development, and democratic engagement. First, policymakers must recognize the growing influence of transnational political actors in shaping electoral outcomes, particularly within diaspora communities. Governments should establish legal frameworks that ensure fair political participation while preventing external influence that could undermine domestic electoral integrity. This includes monitoring foreign funding in political campaigns and regulating digital platforms to curb misinformation and political manipulation. Second, political leaders and organizations must adapt to the evolving nature of digital legitimacy. Third, electoral commissions and civil society groups should come up with plans to better include diaspora communities in political systems. This includes establishing remote voting mechanisms, ensuring that electoral information is accessible in multiple languages, and fostering inclusive political dialogues. Diaspora populations, governments can create more representative and participatory electoral systems toward acknowledging the political agency.

Thailand should adopt policies that encourage greater political participation from its diaspora. Establishing online voting platforms and remote voting centers in Thai embassies abroad can provide greater access to expatriate voters. Chinese community leaders also should implement transparent and accountable procedures in the selecting process of community leaders, including establishing an extensive system of collaboration with the Thai government and local communities to enhance their legitimacy and authority. Furthermore, implement an all-encompassing strategy that takes into account the wide range of cultural and societal values present among the Chinese community in Thailand, which is also inevitable to build reciprocity and social capital among Chinese community members. Therefore, community members should encourage the understanding and appreciation of various Chinese cultural principles during the selection process and in their leadership roles within the community. It also enhances engagement in intercultural collaboration projects to enhance connections with the Thai government and local community and establish enduring legitimacy, which can lead to improved mutual understanding and support for initiatives that benefit both the Chinese community and the Thai society.

### Limitations and future study directions

Despite significant advancements in understanding leadership legitimacy and electoral processes, this study has some limitations. Firstly, this study focuses on the Chinese community in Thailand. Future studies should expand their scope to include comparative analyses of leadership legitimacy in diverse political contexts, such as authoritarian regimes or transitional democracies. Second, while digital legitimacy has been recognized as an emerging factor, there is still a lack of empirical research on how social media platforms and digital technologies specifically shape electoral legitimacy. Future studies should explore the role of social media in shaping public perceptions of leadership credibility. While existing research acknowledges the influence of diaspora communities in elections, there is limited data on how these communities navigate dual loyalties and political identities. Future studies should investigate the impact of transnational political campaigns, cross-border funding, and identity-based mobilization on electoral outcomes. Furthermore, research on leadership legitimacy often lacks interdisciplinary approaches that integrate political science, sociology, and technology studies, which are crucial for understanding the multifaceted nature of legitimacy in contemporary governance. Future scholarship should adopt a more holistic approach, incorporating insights from behavioral sciences, communication studies, and international relations to understand legitimacy dynamics.

## Data Availability

The raw data supporting the conclusions of this article will be made available by the authors, without undue reservation.
